# Efficacy of thalidomide in a girl with inflammatory calcinosis, a severe complication of juvenile dermatomyositis

**DOI:** 10.1186/1546-0096-8-6

**Published:** 2010-02-04

**Authors:** Takako Miyamae, Fumie Sano, Remi Ozawa, Tomoyuki Imagawa, Yoshiaki Inayama, Shumpei Yokota

**Affiliations:** 1 Department of Pediatrics, Yokohama City University, 3-9 Fukuura, Kanazawaku, Yokohama 236-0004, Japan; 2 Department of Pathology, Yokohama City University, 3-9 Fukuura, Kanazawaku, Yokohama 236-0004, Japan

## Abstract

We report a 14-year-old girl with juvenile dermatomyositis (JDM) complicated by severe inflammatory calcinosis successfully treated with thalidomide. She was diagnosed as JDM when she was 4 years old after a few months of increasing lethargy, muscle pain, muscle weakness, and rash. During three months, clinical manifestations and abnormal laboratory findings were effectively treated with oral prednisolone. However, calcinosis was recognized 18 months after disease onset. Generalized calcinosis rapidly progressed with high fever, multiple skin/subcutaneous inflammatory lesions, and increased level of CRP. Fifty mg/day (1.3 mg/kg day) of oral thalidomide was given for the first four weeks, and then the dose was increased to 75 mg/day. Clinical manifestations subsided, and inflammatory markers had clearly improved. Frequent high fever and local severe pain with calcinosis were suppressed. The levels of FDP-E, IgG, and tryglyceride, which were all elevated before the thalidomide treatment, were gradually returned to the normal range. Over the 18 months of observation up to the present, she has had no inflammatory calcinosis, or needed any hospitalization, although established calcium deposits still remain. Her condition became painless, less extensive and less inflammatory with the CRP level below 3.08 mg/dL. Recent examination by whole-body 18F-FDG-PET-CT over the 15 months of thalidomide treatment demonstrated fewer hot spots around the subcutaneous calcified lesions.

## Background

Juvenile dermatomyositis (JDM) is a systemic connective tissue disease characterized by typical skin rash and chronic muscle inflammation of uncertain etiology [1]. Classic JDM presents with an insidious progression of malaise, easy fatigue, muscle weakness, fever, and rash that may predate diagnosis by three to six months. Calcinosis is one of the severe complications of JDM, and despite recent progress in the treatment of this disorder, it still occurs in up to 40% of patients [2,3]. The onset of calcinosis usually occurs one to three years after that of the illness.

Our understanding of the pathogenesis of calcinosis is still very limited. However, it has begun to come into focus through the following recent findings. The calcinosis itself is associated with inflammation. It has been reported that macrophages and proinflammatory cytokines, such as IL-6, IL-1, and TNF-alpha, were present in the white, calcium-rich fluid (calcium milk) collected from a patient [4]. Moreover, calcinosis has been more frequently associated with TNF-alpha-308A promoter polymorphism, which is associated with increased TNF- alpha production by peripheral blood mononuclear cells [5].

The deposition of calcium, mostly in the skin, around the joints, and in the intermuscular fascial planes, may cause more long-term disability than the myositis itself. The relatively low incidence of calcinosis observed in recently reported case series suggests that earlier diagnosis and more aggressive treatment such as corticosteroid and immunosuppressant therapy are required [6]. However, once established, the disease is still difficult to treat.

Recently, the effectiveness of the monoclonal antibody to TNF-alpha, infliximab, in the treatment of refractory juvenile dermatomyositis with calcinosis has been reported [7]. In that study, calcinosis was still present in all five patients who received infliximab, but, notably, it became softer, painless and, in four cases, less widespread.

Thalidomide has been accepted as an immunomodulatory drug in refractory pediatric autoimmune diseases such as Behcet's disease and systemic onset juvenile idiopathic arthritis [8,9]. It has been demonstrated that thalidomide selectively inhibits the TNF-alpha and IL-6 mRNA expression in human peripheral blood mononuclear cells [10]. The inhibitory effect of thalidomide on monocyte TNF-alpha is thought to be the main mechanism of its action as an anti-inflammatory agent [11].

We report a case of severe calcinosis in a 14-year-old girl who was treated with thalidomide, a treatment encouraged by the partial effectiveness of etanercept, a soluble TNF receptor fusion protein.

## Case presentation

**Figure 1:**
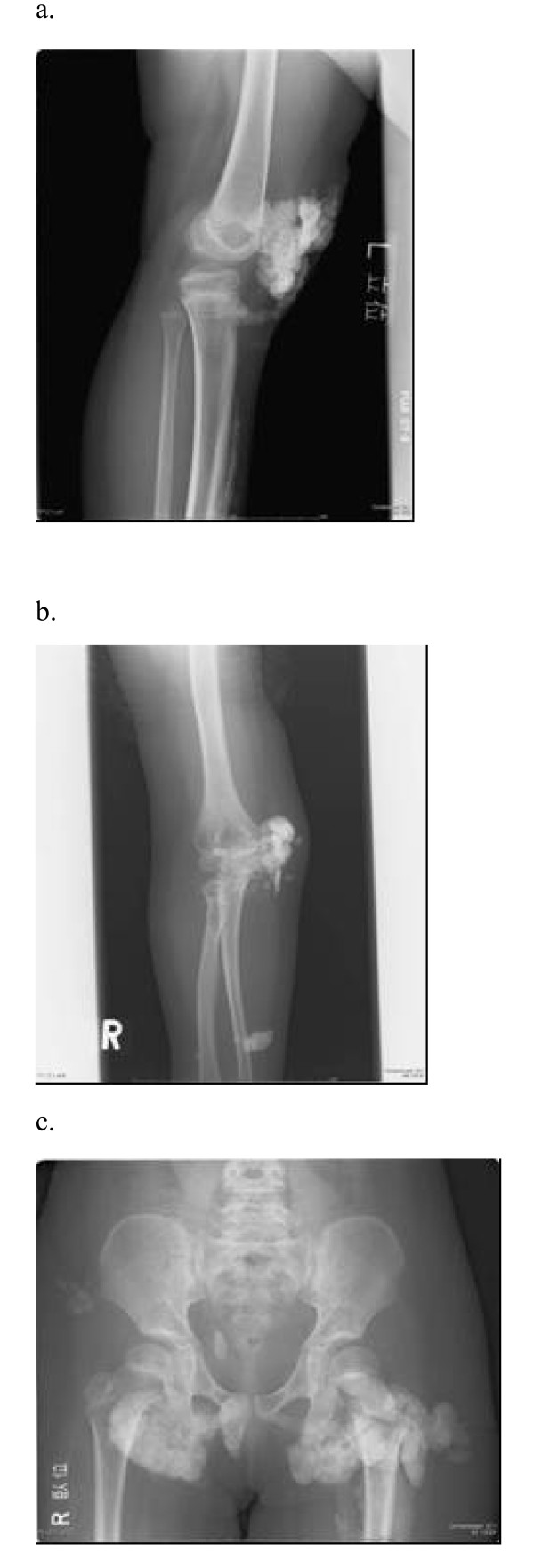
**X-ray findings of extremities and pelvis**. a. Calcium deposit around right knee in April 2001, 3 years and 3 months after JDM onset. b. Subcutaneous calcium nodule over right elbow in January 2003, 5 years after JDM onset. c. Tumoral calcinosis in the buttocks in September 2003, 5 years and 8 months after JDM onset.

**Figure 2:**
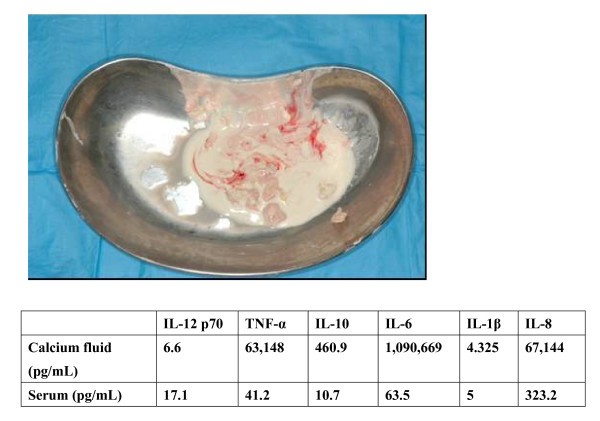
**Calcium milk drained with small amounts of calcium deposits from buttock (top)**. Characteristics of proinflammatory cytokines in serum and calcium milk. (bottom). Examination of subcutaneous calcium milk revealed highly elevated levels of IL-6, TNF-α, and IL-1β.

**Figure 3:**
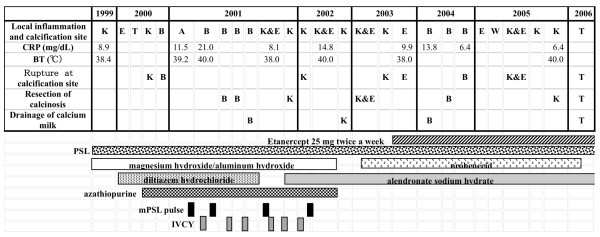
**Clinical course of the patient and "inflammatory calcinosis" defined as subcutaneous inflammation caused by calcification with one or more of: (1) pain (VAS>50 mm), (2) fever (>38.0°), and (3) elevated level of CRP (>5 mg/dL)**. K: knee, E: elbow, T: thigh, B: buttock, W: wrist, mPSL: methyl prednisolone; IVCY:intravenous cyclophosphamide pulse.

**Figure 4:**
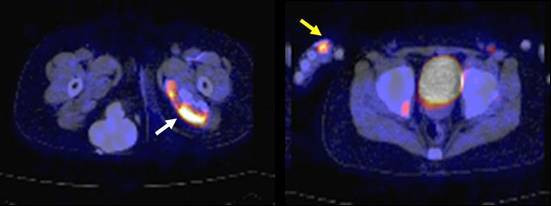
**Findings of 18 FDG-PET/CT showing "inflammatory calcinosis"in August 2006**. Subcutaneous and intramuscular calcification were recognized as in conventional plain CT. Hot spots (white arrow) along with intramuscular calcification indicate inflammation. Even the small lesion of inflammation around a right digit (yellow arrow) can be seen clearly.

**Figure 5:**
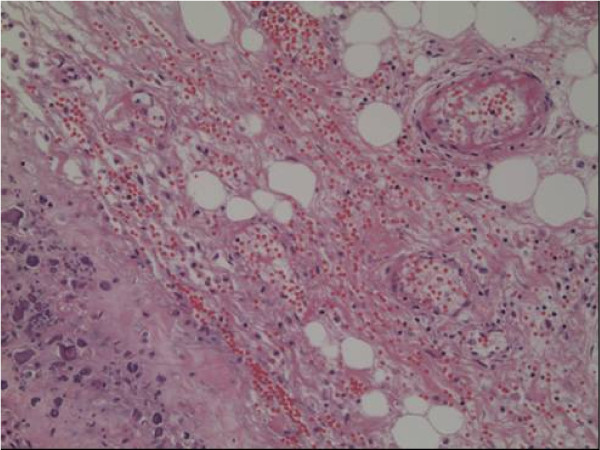
**Light microscopy of calcium deposits with soft tissue obtained in the left thigh during surgery**. Bordered with calcium plaques (arrow), fibrinoid vasculitis was observed with hemorrhage and degeneration of adipose cells. (H&E stain, 20×).

The patient was a girl of 14 years of age, in whom JDM had been diagnosed in January 1998, when, at 4 years of age, she had been experiencing increasing lethargy, muscle pain, muscle weakness, and rash for a few months. Calcinosis had been recognized 18 months after the diagnosis was made (Fig. 1). For 3 months, clinical manifestations and abnormal laboratory findings were effectively treated with oral prednisolone. Her muscle weakness and elevated muscle-derived enzymes were normalized with this treatment, and the myositis was stable after that. However, generalized calcinosis progressed rapidly with high fever, multiple skin and subcutaneous inflammatory lesions, and an increased level of CRP. Examination of the subcutaneous calcium milk revealed markedly elevated levels of IL-6, TNF-alpha, and IL-1beta by ELISA (Fig 2). Methylprednisolone pulses, cyclophosphamide, cyclosporine, azathioprine, probenecid and magnesium hydroxide and aluminum hydroxide were administered, but these treatments failed, resulting in repeated rupture, drainage and resection at calcinosis sites (Fig 3). "Inflammatory calcinosis" events, defined as subcutaneous inflammation caused by calcification with one or more of the following, (1) pain (VAS>50 mm), (2) fever (>38.0°C, and (3) an elevated level of CRP (>5 mg/dL), were not suppressed by the conventional treatments at all. As shown in Fig. 4, fusion imaging systems combining 18F-FDG PET (Fluorodeoxyglucose-Positron Emission Tomography) and CT visualized anatomical location of the hot spot lesions of inflammatory calcinosis. Pathological evaluation of this inflammatory calcinosis revealed calcium plaques with fibrinoid vasculitis, inflammatory cell infiltration, hemorrhage and degeneration of adipose cells. (Fig. 5)

Infliximab, the monoclonal antibody to TNF-alpha, was administered in 2003, when the patient was 9 years old, but it was discontinued due to adverse effects and replaced with etanercept. Being encouraged by the partial effectiveness of subcutaneous injection of 25 mg (0.8 mg/kg) of etanercept twice a week, her high fevers became less frequent. However, surgical approaches to the removal of the calcium deposits were still required because of inflammatory calcinosis with severe pain, rupture, or both at the calcinosis sites.

Etanercept treatment was replaced with thalidomide treatment in 2006 at the age of 12 after receiving approval from the ethical committee and informed consent from the patient and her parents. Fifty mg/day (1.3 mg/kg day) of oral thalidomide (Sauramide^®^, Penn Pharmaceuticals, Tredegar, UK) was given for the first four weeks, and then the dose was increased to 75 mg/day. Clinical manifestations subsided, and inflammatory markers had clearly improved (Table 1). Frequent high fever and local severe pain with calcinosis were suppressed. The levels of FDP-E, IgG, and tryglyceride, which were all elevated before the thalidomide treatment, were gradually returned to the normal range. Over the 18 months of observation up to the present, she has had no inflammatory calcinosis, or needed any hospitalization, although established calcium deposits still remain. Her condition became painless, less extensive and less inflammatory with the CRP level below 3.08 mg/dL. Recent examination by whole-body 18F-FDG-PET-CT over the 15 months of thalidomide treatment demonstrated fewer hot spots around the subcutaneous calcified lesions.

**Table 1:** Laboratory findings of the patient.

	2001	2006		
	During inflammatory calcinosis	Before thalidomide (inacitive phase)	3 months	12 months
WBC (μL)	27,300	7,100	6,400	5,200

Hb (g/dL)	11.6	11.3	9.5	12.7

Platelets (×10^4^/μL)	24.7	24.6	35.7	35.7

				

ESR (mm/h)	27	17	3	4

				

FDP-E (ng/mL)	225	237	95	85

				

AST (U/L)	20			

ALT (U/L)	7			

LDH (U/L)	271			

CRP (mg/dL)	21.0	0.2	0.1	0.4

Triglycerides (mg/dL)	nd	251	74	93

Total cholesterol (mg/dL)	nd	121	145	156

				

IgG (mg/dL)	nd	2,056	813	916

The levels of FDP-E, IgG, and triglycerides were all elevated before thalidomide gradually reduced them to the normal range.

## Discussion

Treatment with thalidomide produced a sustained major clinical improvement in a case of JDM with inflammatory calcinosis refractory to the conventional corticosteroids and immunosuppressants and even to etanercept.

The mechanism of the inflammatory calcinosis of JDM is still unclear. Pachman *et al*. speculated that calcification occurring at sites where it is undesirable, as opposed to physiologic calcification, is usually designated as "pathologic" or, when associated with cell death, as "dystrophic" in nature [12]. Scientifically, at least, as Table 1 shows, it has been shown that inflammation accompanied by calcinosis is characterized by multiple proinflammatory cytokinemia including TNF-alpha, IL-6, and IL-1beta. The level of IL-10 in the calcium milk was also elevated. IL-10 is a cytokine with potent anti-inflammatory properties, which represses the expression of inflammatory cytokines such as TNF-alpha, IL-6 and IL-1 by activated macrophages. This finding may indicate that in parallel with the predicted activation of the immune response and tissue injury pathways caused by TNF-alpha, IL-6 and IL-1, there is simultaneous activation of pathways for the counter-regulatory and the protective mechanisms that would balance and limit the ongoing inflammatory/immune responses.

The effect of infliximab in refractory calcinosis with juvenile dermatomyositis was reported previously [7]. The anti-TNF-alpha biologics such as infliximab and etanercept are designed specifically to target TNF-alpha. Unlike the mechanism of action of the biologics, thalidomide has complex immunomodulatory and anti-inflammatory properties. It has been shown to downregulate the production of TNF-alpha and other proinflammatory cytokines, to inhibit the transcription factor nuclear factor kappa B (NF kappaB), and to downregulate cyclooxygenase 2 [13,14]. This might explain why thalidomide was more effective in the present case than etanercept. The calcified deposits contained the bone proteins osteopontin, osteonectin, and sialoprotein. Hydroxyapatite was the only mineral detected, but the tissue was distinct from bone, with an extremely high mineral content and an irregular distribution of minerals [12]. The same authors also reported that calcifications from JDM patients contained more osteonectin than is usually found in human bone. In vitro studies indicate that osteonectin can bind collagen and regulate angiogenesis, metalloproteinase expression, cell proliferation, and cell-matrix interactions [15].

Thalidomide is not only an immunomodulatory drug but also has anti-angiogenic effects [16]. It has been reported, for example, in myeloma, to suppress angiogenic factors such as vascular endothelial growth factor (VEGF), and inflammatory genes such as TNF-alpha and IL-6 [17,18]. The anti-angiogenic effect may be a part of the thalidomide mechanism that reverses inflammatory calcinosis.

The pathological specimen showed fibrinoid vasculitis surrounding calcinosis. It is hard to explain whether the inflammatory calcinosis is due to muscle vasculitis because there was no muscle vasculitis where no calcinosis was present. Muscle vasculitis arising from JDM should be symmetrical. We prefer to surmise that fibrinoid vasculitis was followed by the calcinosis universalis itself. Besides, no report of a direct relationship between vasculitis and calcinosis universalis has appeared in the literature.

The findings of 18F-FDG-PET-CT after thalidomide treatment included fewer hot spots showing local inflammation around subcutaneous calcified lesions. This suggested that the subcutaneous pooling of calcium milk may be the cause of proinflammatory cytokinemia and subsequent "inflammatory calcinosis" in JDM, and the suppression of NF kappaB activation by thalidomide is likely to be beneficial for inhibiting the systemic spread of inflammation.

## Consent

Written informed consent was obtained from the patient for publication of this case report and any accompanying images. A copy of the written consent is available for review by the Editor in Chief of this journal.

## Competing interests

The authors declare that they have no competing interests.

## Authors' contributions

TM drafted the manuscript and participated in its design. SF, RO, and TI participated in drafting of the manuscript and participated in its design. YI participated in the drafting of the manuscript and supplied the pathological image used for the manuscript. SY conceived of the case report, participated in drafting the manuscript and gave final approval for the version to be submitted for publication.
